# Maternal and perinatal outcomes of pregnant women with echocardiographically high probability of pulmonary arterial hypertension

**DOI:** 10.1002/ijgo.71119

**Published:** 2026-06-18

**Authors:** Laura Belmont‐Rojo, Rosa Hernandez‐Cruz, Ana Jimena Lopez‐Diaz, Diana Yazmin Copado‐Mendoza, Tirso Rubalcava‐Rubalcava, Mario Isaac Lumbreras‐Marquez, Maria Antonia Pijuan‐Domenech

**Affiliations:** ^1^ Cardiology Division Instituto Nacional de Perinatologia Mexico City Mexico; ^2^ Maternal Fetal Medicine Division Clinic Hospital Barcelona Spain; ^3^ Maternal‐Fetal Medicine Division Instituto Nacional de Perinatologia Mexico City Mexico; ^4^ Head of the Cardiology Division Centro Médico Licenciado Adolfo López Mateos Toluca Mexico; ^5^ Epidemiology and Public Health Division Universidad Panamericana School of Medicine Mexico City Mexico; ^6^ CIBER‐CV A Coruña Spain; ^7^ Integrated Vall Hebron‐Sant Pau Adult Congenital Heart Disease Hospital Vall d'Hebron Barcelona Spain; ^8^ Department of Cardiology Hospital Vall d'Hebron Barcelona Spain

**Keywords:** cardiac complications, heart failure, perinatal outcomes, pregnancy, pulmonary arterial hypertension

## Abstract

**Objective:**

This study aimed to describe maternal and perinatal outcomes among pregnant women with echocardiographically high probability of pulmonary arterial hypertension (PAH) managed at a quaternary center and to compare outcomes between women with and without cardiac complications (CC).

**Methods:**

This retrospective cohort study included pregnant women with echocardiographically estimated high probability of PAH and managed at a referral center between 2015 and 2022. Clinical, obstetric, and neonatal outcomes were obtained from medical records. CC were defined as heart failure, pulmonary edema, arrhythmia, pulmonary embolism, or maternal death during pregnancy or up to 6 weeks postpartum. Outcomes were compared between women with and without CC. Receiver operating characteristic (ROC) curve analysis was performed as an exploratory analysis to assess the discriminatory ability of pulmonary artery systolic pressure (PASP) for CC.

**Results:**

Sixty‐two pregnancies in 60 women (mean age 24.5 ± 6.5 years) were analyzed. Eight pregnancies (12.9%) were electively terminated, while 54 (87.1%) continued. CC occurred in 11 pregnancies (17.7%), mainly heart failure. Four maternal deaths (6.5%) were recorded. Among ongoing pregnancies, the median gestational age at delivery was 37.1 weeks, and the median birthweight was 2445 g. Fetal growth restriction occurred in 27.7%, and 62.9% of deliveries were by cesarean section. Neonatal mortality was 5.5%. In exploratory analysis, a PASP threshold of 64 mmHg predicted CC with 91% sensitivity and 60% specificity (area under the curve = 0.749).

**Conclusion:**

Pulmonary arterial hypertension is a high‐risk condition in pregnancy, with substantial maternal and perinatal complications. Although maternal mortality was lower than historically reported in similar settings, the incidence of CC remained considerable. PASP might reflect disease severity and shows exploratory discriminatory ability but requires cautious interpretation.

## 1 INTRODUCTION

Pulmonary arterial hypertension (PAH) represents a major risk factor for severe maternal complications during pregnancy and has historically been associated with unacceptably high maternal mortality rates.^1^ In healthy women, the physiological adaptations of pregnancy, including increased plasma volume, elevated cardiac output, and decreased systemic vascular resistance, are generally well tolerated. However, in women with PAH, these hemodynamic changes can critically compromise cardiovascular function. Pregnancy‐induced volume overload and reduced systemic vascular resistance can exacerbate the underlying pathology of PAH. The inability of the pulmonary circulation to accommodate increased cardiac output leads to elevated pulmonary artery and right heart pressures, often culminating in cardiac decompensation.^2^, ^3^

Pulmonary arterial hypertension is estimated to affect approximately 9.7 per 100 000 women,^4^ and nearly 80% of affected individuals are of reproductive age.^5^ Although PAH is rare in pregnancy, it carries a disproportionately high maternal mortality rate. Earlier studies have reported mortality rates as high as 30%–56% in pregnant women with PAH when advanced therapies are unavailable.^1^, ^6^ In addition to maternal morbidity, PAH in pregnancy is associated with adverse perinatal outcomes, including high rates of preterm birth (85%–100%), fetal growth restriction (FGR) (3%–33%), and fetal or neonatal death (7%–13%). Moreover, clinical management decisions (e.g., timing and mode of delivery, anesthetic management, and the use of uterotonic agents) might further influence maternal and neonatal outcomes in this high‐risk population.^7^

Despite PAH being classified as modified World Health Organization (mWHO) 2.0 functional class IV (indicating that pregnancy carries very high maternal and fetal risk), some women with PAH still choose to conceive or to continue unplanned pregnancies.^8^ Recent evidence suggests that outcomes might differ according to PAH subtype, with the highest mortality observed in women with Eisenmenger syndrome and non‐vasoreactive idiopathic PAH.^9^, ^10^ Outcomes also appear to vary with disease severity. Although no safe cut‐off exists for elevated pulmonary artery systolic pressure (PASP) and associated risk, pregnancies with mild PAH appear to have better outcomes. Nevertheless, reclassification of mild PAH into mWHO 2.0 type III has not been considered.^7^, ^11^

This study aimed to describe maternal and perinatal outcomes in pregnant women with echocardiographically high probability of PAH managed at a tertiary academic center. We also compared outcomes between those who experienced cardiac complications (CC) during pregnancy or the postpartum period and those who did not.

## 2 MATERIALS AND METHODS

### 2.1 Study design and population

This single‐center retrospective cohort study was conducted at the Instituto Nacional de Perinatologia, a quaternary center in Mexico City, from November 2015 to January 2022. All consecutive pregnant women with echocardiographically high probability of pulmonary hypertension (PAH) who received prenatal care at the institution and either underwent pregnancy termination or were managed through delivery were included. No cases were excluded due to incomplete medical records, as data were complete for all cases with respect to the exposure and cardiac outcomes of interest; therefore, complete‐case analysis was applied.

The diagnosis of PAH in this study was based primarily on echocardiographic estimation of pulmonary artery systolic pressure (PASP). Although right heart catheterization remains the gold standard for the diagnosis of PAH, it was not performed during pregnancy in this cohort because of its invasive nature. PASP was estimated via tricuspid regurgitation velocity, provided there was no pulmonary stenosis or other right ventricular outflow obstruction. In patients with structural heart disease, including ventricular septal defect and patent ductus arteriosus, PASP was estimated by echocardiography using the maximum pressure gradient across the defect and noninvasive systemic arterial pressure, according to standard echocardiographic practice. For study purposes, PAH severity was classified as mild (pulmonary artery systolic pressure 36–55 mmHg) or severe (> 55 mmHg) using echocardiographic thresholds, given the absence of invasive hemodynamic assessment in this cohort. Eisenmenger syndrome was defined as PAH in the presence of bidirectional shunt. Echocardiographic parameters used for analysis corresponded to the evaluation performed at the time of the patient's initial assessment at our institution during pregnancy; gestational age at echocardiographic assessment was recorded in the database.

All echocardiograms were performed by cardiologists using standardized protocols and Philips Affinity 50 equipment.

### 2.2 Data collection

Trained researchers used a standardized data abstraction form to extract data from medical records, which were then reviewed by a senior investigator for accuracy. Collected variables included demographics, obstetric history, PAH etiology (idiopathic, congenital heart disease [CHD]‐associated, connective tissue disease‐associated, or other), WHO functional class, use of pulmonary vasodilator therapy (e.g., prostanoids [iloprost], endothelin receptor antagonists [bosentan], phosphodiesterase inhibitors [sildenafil]), and New York Heart Association (NYHA) functional class.^11^ Researchers also retrospectively calculated the modified mWHO 2.0 classification (all patients included in group IV), NYHA, and CARPREG II^12^, ^13^ scores for all patients, considering pre‐pregnancy status.

Echocardiographic parameters included PASP, right ventricular function, right atrial dilation, and pericardial effusion as well as complete echocardiographic assessment as per current guidelines.^14^ Obstetric data included gestational age at delivery, mode of delivery, type of anesthesia, estimated blood loss, hypertensive disorders, and FGR. FGR was defined according to Delphi consensus criteria: estimated fetal weight (EFW) or abdominal circumference (AC) < 3rd percentile, or EFW/AC < 10th percentile with abnormal Doppler findings or declining growth centiles. FGR was further classified as early onset when diagnosed before 32 weeks of gestation and as late onset when diagnosed at or after 32 weeks.^15^ Neonatal data included birthweight, 5‐min Apgar scores, neonatal intensive care unit (NICU) admission, and perinatal mortality.

### 2.3 Study outcomes

The primary outcome was the description of maternal and perinatal outcomes. Maternal outcomes included management during pregnancy and the puerperium, associated comorbidities (e.g., hypertensive disorders, systemic lupus erythematosus, antiphospholipid syndrome, hypothyroidism, and obesity), and CC. A composite CC outcome was used to capture overall maternal cardiovascular decompensation during pregnancy and the puerperium and to allow analysis in the context of a limited sample size and relatively infrequent individual events. CC were defined as a composite of heart failure requiring treatment or hospitalization, arrhythmia requiring medical intervention, thromboembolic events, acute pulmonary edema, and maternal death, classified as early (within 42 days postpartum) or late (43 days to 1 year postpartum) due to cardiovascular causes. CC were identified through review of electronic medical records and adjudicated based on documented clinical diagnoses and treatment requirements during pregnancy or the puerperium. Perinatal outcomes included gestational age at delivery, mode of delivery, FGR, blood loss, birthweight, Apgar score at 5 min, NICU admission, and mortality. The secondary outcome was the comparison of maternal and perinatal outcomes according to the presence or absence of maternal CC.

### 2.4 Statistical analysis

Continuous variables were assessed for normality using histograms and reported as mean ± standard deviation or median with interquartile range, as appropriate. Categorical variables were reported as frequencies and percentages (%). For comparative analyses, patients were retrospectively stratified according to the presence or absence of maternal CC during pregnancy or the puerperium. Comparisons between women with and without CC were made using the *χ* ^2^ or Fisher's exact test for categorical variables and Student's *t*‐test or Mann–Whitney *U*‐test for continuous variables. Standardized differences were used to compare baseline characteristics, with imbalance defined as an absolute value > 0.20.

As an exploratory analysis, we evaluated the predictive value of PASP for maternal CC using receiver operating characteristic (ROC) curve analysis. The area under the curve (AUC), sensitivity, specificity, and the optimal threshold (determined by Youden's index) were reported. Given the limited number of cardiovascular events in our cohort, multivariable modeling was not performed because of the high risk of overfitting; therefore, the association between PASP and CC should be interpreted cautiously, and independent predictive value cannot be established. A *P*‐value < 0.05 was considered statistically significant. Statistical analyses were performed using Stata version 19.0 (StataCorp, College Station, TX, USA).

### 2.5 Ethical considerations

The investigation conforms with the principles outlined in the Declaration of Helsinki.^16^ This study was approved by the Research Ethics Committee of the Instituto Nacional de Perinatologia (CEI‐RETRO‐09‐2025). Due to the retrospective nature of the study, the requirement for informed consent was waived.

## 3 RESULTS

A total of 62 pregnancies in 60 women with echocardiographically high probability of PAH were included. The mean maternal age was 24.5 ± 6.5 years, and nearly all women were nulliparous (96.8%) with singleton pregnancies. The maternal mortality rate was 6.5% (four deaths), and CC occurred in 11 cases (17.7%). Most cases (88.7%) of women had PAH associated with CHD, and 11.3% had acquired PAH. Baseline maternal characteristics are presented in Table 1. Comorbidities were uncommon; however, hypothyroidism was more frequent in women who developed CC (18.2% vs. 2.0%). The mean body mass index was 23.6 ± 3.6 kg/m^2^, with no clinically significant differences between groups.

**TABLE 1:** Demographic characteristics of the study population.

Parameter	Full cohort	Without cardiac complications	With cardiac complications	Standardized difference*
	*N* = 62	*N* = 51	*N* = 11	
Age, years, mean, SD	24.45 ± 6.51	24.65 ± 6.42	23.55 ± 7.16	0.161
Pregestational diabetes, *n* (%)	0 (0)	0 (0)	0 (0)	—
BMI, kg/m^2^, mean, SD	23.59 ± 3.56	23.81 ± 3.58	22.57 ± 3.44	0.353
Weight classification, *n* (%)				0.350
Underweight	5 (8.1)	3 (5.9)	2 (18.2)	
Normal weight	37 (59.7)	31 (60.8)	6 (54.5)	
Overweight	17 (27.4)	14 (27.5)	3 (27.3)	
Obesity class I	3 (4.8)	3 (5.9)	0 (0.0)	
Obesity class II	—	—	—	
Obesity class III	—	—	—	
Chronic hypertension, *n* (%)	4 (6.5)	4 (7.8)	0 (0)	0.408
Systemic lupus erythematosus, *n* (%)	1 (1.6)	1 (2.0)	0 (0)	0.198
Antiphospholipid syndrome, *n* (%)	0 (0)	0 (0)	0 (0)	—
Hypothyroidism, *n* (%)	3 (4.8)	1 (2.0)	2 (18.2)	−0.535
Recurrent pregnancy loss, *n* (%)	2 (3.2)	2 (3.9)	0 (0)	0.282
Preeclampsia history, *n* (%)	0 (0)	0 (0)	0 (0)	—
Congenital heart disease family history, *n* (%)	4 (6.5)	4 (7.8)	0 (0)	0.408
Gravidity, median, IQR	1.0 [1.0, 2.0]	1.0 [1.0, 2.0]	1.0 [1.0, 2.0]	0.525
Pregestational weight, kg, mean, SD	57.1 [50.5, 60.9]	59.0 [50.5, 63.0]	55.0 [45.4, 57.2]	0.735

Abbreviations: BMI, body mass index; IQR, interquartile range; SD, standard deviation.

* Standardized differences were used to compare baseline characteristics; an absolute standardized difference >0.20 was considered indicative of imbalance.

### 3.1 Medication use

As shown in Table S1, pulmonary vasodilators and diuretics were rarely used before pregnancy (only one patient received sildenafil) but were more frequently administered during pregnancy and the puerperium, particularly in women with CC. These therapies were generally initiated in response to clinical deterioration and are, therefore, presented descriptively as markers of escalation of care rather than predictors of CC. In the puerperium, 81.8% of women with CC received pulmonary vasodilators compared to 31.4% of those without complications (*P* = 0.005). Two patients were treated with iloprost, one with bosentan, and the remaining patients received sildenafil. All patients with CC received diuretics (100% vs. 5.9%, *P* < 0.001). Beta‐blockers and inotropes were also used more frequently in the CC group (*P* < 0.001).

### 3.2 Cardiac risk stratification and echocardiographic findings

As shown in Table 2, baseline NYHA functional class was worse among women who developed CC, with 72.7% classified as NYHA II compared to 3.9% in those without complications (*P* < 0.001). By definition, all women were classified as mWHO 2.0 risk class IV. The median CARPREG II score was significantly higher in the group with CC (6 [5, 9] vs. 4 [2, 6], *P* = 0.007) The left ventricular ejection fraction (LVEF) was lower in those with CC (50% [47, 58] vs. 60% [56, 66], *P* = 0.002), and PASP was markedly elevated (96 mmHg [66, 102] vs. 54.5 mmHg [45, 78], *P* = 0.010). Eisenmenger syndrome was present in four patients (6.5%), of whom 50% experienced CC.

**TABLE 2:** Cardiologic features of the study population.

Parameter	Full cohort	Without cardiac complications	With cardiac complications	*P*
	*N* = 62	*N* = 51	*N* = 11	
NYHA classification, *n* (%)				< 0.001
I	51 (82.3)	49 (96.1)	2 (18.2)	
II	10 (16.1)	2 (3.9)	8 (72.7)	
III	—	—	—	
IV	1 (1.6)	0 (0)	1 (9.1)	
Modified WHO 2.0. Classification of Maternal Cardiovascular Risk, *n* (%)				—
IV	62 (100)	51 (100)	11 (100)	
CARPREG II score, median, IQR	4 [2, 6]	4 [2, 6]	6 [5, 9]	0.007
ZAHARA score, median IQR	0.8 [0.0, 1.5]	0.0 [0.0, 1.5]	1.5 [0.0, 1.8]	0.070
Gestational age for transthoracic echocardiogram, weeks, median, IQR	28.0 [20.3, 33.5]	28.0 [18.6, 35.0]	26.2 [22.0, 28.0]	0.280
Left ventricular ejection fraction, median, IQR	58 [55, 65]	60 [56, 66]	50 [47, 58]	0.002
Tricuspid annular plane systolic excursion, median, IQR	21 [18, 23]	21 [18, 25]	20.5 [17, 23]	0.670
Pulmonary artery systolic pressure on echocardiogram, median, IQR	64 [48, 90]	54.5 [45, 78]	96 [66, 102]	0.010
Acquired heart disease, *n* (%)	7 (11.3)	6 (11.8)	1 (9.1)	0.999
Congenital heart disease, *n* (%)	55 (88.7)	45 (88.2)	10 (90.9)	0.999
Prosthesis or pacemaker, *n* (%)	3 (4.8)	3 (5.9)	0 (0.0)	0.999
Arrhythmia history, *n* (%)	3 (4.8)	3 (5.9)	0 (0.0)	0.999
Eisenmenger syndrome, *n* (%)	4 (6.5)	2 (3.9)	2 (18.2)	0.141

Abbreviations: CAPREG, Cardiac Disease in Pregnancy; IQR, interquartile range; NYHA, New York Heart Association; SD, standard deviation.

### 3.3 Obstetric and perinatal outcomes

Of the 62 pregnancies, eight (12.9%) ended in pregnancy termination, with a median gestational age of 13.0 weeks [8.5, 16.5]. The remaining 54 pregnancies (87.1%) continued. As shown in Table 3, FGR was diagnosed in 15 cases (27.7%). The median gestational age at delivery was 37.1 weeks [36.0, 38.8], and the median birthweight was 2445 g [2050, 2795], with no significant differences between groups. C‐section was the most common mode of delivery (63.0%). Estimated blood loss was significantly higher in women with CC (500 mL [300, 600] vs. 300 mL [150, 400], *P* = 0.014). Two cases of obstetric hemorrhage occurred in forceps‐assisted vaginal deliveries. General anesthesia was used more frequently in the CC group (36.4% vs. 5.9%, *P* = 0.015), while neuraxial anesthesia was less common (54.5% vs. 92.2%, *P* = 0.006). NICU admission occurred in three neonates (5.5%), with a trend toward more frequent and longer NICU stays among those born to mothers with CC (median length of stay 7.5 days [5, 17] vs. 3 days [2, 7], *P* = 0.036). Neonatal mortality occurred in three cases (5.5%).

**TABLE 3:** Obstetric and neonatal outcomes.

Parameter	Full cohort	Without cardiac complications	With cardiac complications	*P*
	*N* = 62	*N* = 51	*N* = 11	
^a^Gestational age for last scan, weeks, median, IQR	33.8 [32, 36]	34 [32, 36.3]	32.4 [29.3, 33.5]	0.090
^a^Estimated fetal weight for last scan (g), median, IQR	2116 [1800, 2598]	2205 [1819, 2622]	1826 [1100, 2124]	0.030
^a^Estimated fetal weight centile, median, IQR	23 [3, 45.5]	24 [6, 48]	7 [2, 30]	0.230
^a^Fetal growth restriction, *n* (%)	15 (27.7)	11 (25.0)	4 (40.0)	0.419
Early	5 (33.3)	2 (18.18)	3 (75.0)	
Late	10 (66.67)	9 (81.82)	1 (25.0)	
^a^Delivery gestational age, weeks, median, IQR	37.1 [36, 38.8]	37.1 [36.4, 39]	36 [33, 38.4]	0.090
Mode of delivery, *n* (%)				0.896
^a^Vaginal delivery	10 (18.5)	9 (20.4)	1 (10.0)	
^a^Cesarean delivery	34 (63.0)	26 (59.0)	8 (80.0)	
^a^Forceps	10 (18.5)	9 (20.4)	1 (10.0)	
Dilation and evacuation	1 (1.6)	1 (2.0)	0 (0)	
Dilation and curettage	7 (11.3)	6 (11.8)	1 (9.1)	
Blood loss, mL, median, IQR	300 [150, 400]	300 [150, 400]	500 [300, 600]	0.014
Anesthesia mode, *n* (%)				
Neuraxial anesthesia	53 (85.5)	47 (92.2)	6 (54.5)	0.006
General anesthesia	7 (11.3)	3 (5.9)	4 (36.4)	0.015
Sedation	—	—	—	—
^a^Birth weight (g), median, IQR	2445 [2050, 2795]	2522.5 [2225, 2900]	2000 [1090, 2535]	0.070
^a^Height, cm, median, IQR	47 [44, 49]	47 [44, 49]	46.5 [43, 48]	0.330
^a^5‐min Apgar, median, IQR	9 [9, 9]	9 [9, 9]	9 [8, 9]	0.070
^a^Silverman Andersen score, median, IQR	1.5 [1, 2]	1 [1, 2]	2 [1, 2.5]	0.210
^a^NICU admission, *n* (%)	3 (5.5)	1 (2.3)	2 (20.0)	0.079
^a^Neonatal LOS, days, median, IQR	3 [2, 7]	3 [2, 7]	7.5 [5, 17]	0.036
Maternal ICU admission, *n* (%)	39 (62.9)	31 (60.8)	8 (72.7)	0.516
Maternal LOS, days, median IQR	4 [2, 7]	4 [2, 6]	7 [4, 11]	0.070

Abbreviations: ICU, intensive care unit; IQR, interquartile range; LOS, length of stay; NICU, neonatal intensive care unit.

^a^ *N* = 54, excluding patients with termination of pregnancy.

### 3.4 Maternal cardiac complications and mortality

As detailed in Tables S2 and 4, heart failure was the most common complication, occurring in all 11 women with cardiac events (17.7% of the total cohort). Other complications included acute pulmonary edema (4.8%), arrhythmia (1.6%), and pulmonary embolism (1.6%). All four maternal deaths were preceded by heart failure. Three deaths occurred in the early postpartum period (within 7 days), and one occurred during pregnancy, requiring a perimortem cesarean section at 31.5 weeks, which also resulted in neonatal death. Heart failure and maternal death are presented separately in Table 4, in addition to the composite CC outcome, to provide greater clinical transparency regarding the pattern of adverse events in the cohort.

**TABLE 4:** Maternal outcomes during pregnancy or in the puerperium period.

Outcome	Full cohort
	*N* = 62
Heart failure, *n* (%) (95% CI)^a^	11 (17.7) (9, 30)
Acute pulmonary edema, *n* (%) (95% CI)^a^	3 (4.8) (1, 10)
Arrhythmia, *n* (%) (95% CI)^a^	1 (1.6) (0.04, 9)
Pulmonary embolism, *n* (%) (95% CI)^a^	1 (1.6) (0.04, 9)
Maternal death, *n* (%) (95% CI)^a^	4 (6.5) (2, 20)
Composite outcome, *n* (%)^b^ (95% CI)^a^	11 (17.7) (9, 30)
Referral during pregnancy, *n* (%) (95% CI)^a^	9 (14.5) (7, 30)
Referral in the puerperium period, *n* (%) (95% CI)^a^	2 (3.2) (0.4, 10)

Abbreviation: CI, confidence interval.

^a^ Clopper–Pearson method.

^b^ Heart failure, acute pulmonary edema, arrhythmia, pulmonary embolism, maternal death.

### 3.5 Exploratory analysis of pulmonary artery systolic pressure predictive value

In the exploratory analysis, the area under the ROC curve for PASP in predicting maternal CC was 0.749 (95% CI: 0.593–0.904), indicating acceptable discriminatory ability (Figure 1). The PASP threshold of 64 mmHg provided the optimal balance of sensitivity and specificity, yielding a sensitivity of 91% and a specificity of 60%. This value should be interpreted as hypothesis‐generating and not as a validated or clinically actionable threshold. In a sensitivity analysis restricted to pregnancies with CHD‐associated pulmonary hypertension, PASP showed similar discriminatory performance for maternal CCs, with an AUC of 0.733 (95% CI: 0.565–0.901). Given the limited number of events, these subgroup findings should also be interpreted as exploratory.

**FIGURE 1:**
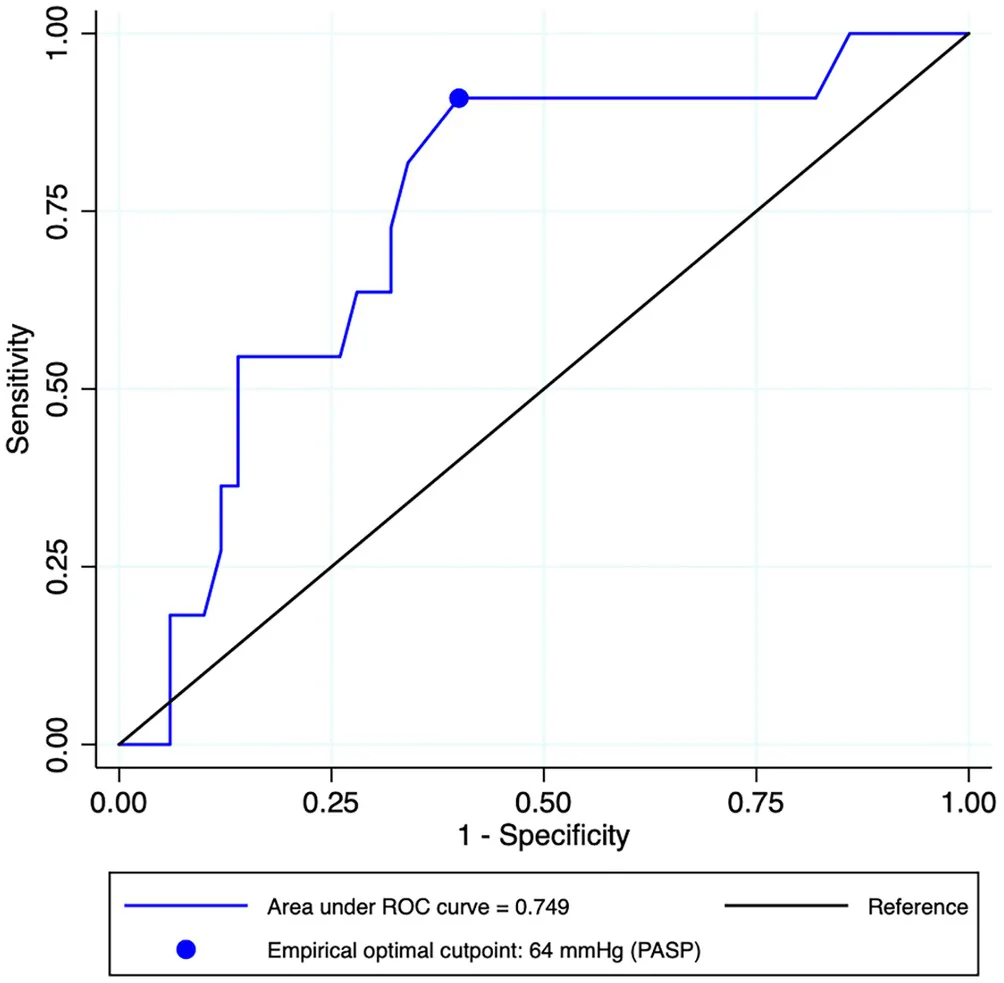
Receiver operating characteristic (ROC) curve for predicting maternal cardiac complications using the pulmonary artery systolic pressure (PASP).

In a comparative ROC analysis for prediction of maternal CCs, as mentioned above, PASP showed an AUC of 0.749 (95% CI 0.593–0.905), whereas CARPREG II showed an AUC of 0.758 (95% CI 0.604–0.913). The difference between the two AUCs was not statistically significant (DeLong *P* = 0.937).

## 4 DISCUSSION

### 4.1 Maternal mortality and cardiovascular complications

Pulmonary arterial hypertension remains a major threat to maternal health during pregnancy. In our cohort, maternal deaths and CCs were observed, with the frequency of these adverse events consistent with recent studies reporting mortality rates between 5% and 12%, often depending on disease severity and the level of care.^5^, ^16^, ^17^ For example, Ladouceur et al. reported a 5% mortality rate in women with CHD‐associated PAH,^18^ while Zhang et al. found 5.7% mortality among women with moderate‐to‐severe PAH.^19^ Likewise, in a systematic review including 272 pregnancies, Low et al. reported a pooled maternal mortality rate of 12%, highest among women with idiopathic PAH (20%) and lower in those with congenital PAH (11%).^20^ Our findings are in line with these contemporary data and suggest that outcomes have improved in specialized centers. We observed that most maternal deaths occurred during the early postpartum period, highlighting this as the most vulnerable phase, as described in other series and demonstrated in a review by Low et al.^20^ This finding underscores the need for prolonged, intensive monitoring in the postpartum intensive care setting, where the careful and thoughtful use of diuretics is necessary to counteract volume overload resulting from uterine contraction‐induced autotransfusion and fluid shifts into the circulation during the puerperium, as well as from fluid and blood administration in cases of bleeding. In our series, no mortality occurred during termination of pregnancy; however, it remains a high‐risk procedure in which a multidisciplinary approach is essential.

Notably, the reported composite CC outcome should be interpreted with caution, as all maternal deaths in our cohort were preceded by heart failure. This temporal and clinical overlap reduces the independence of the composite components and might limit the interpretability of the composite endpoint. Moreover, in the present study, a PASP value of 64 mmHg was identified as the point with the best balance of sensitivity and specificity in this cohort for predicting maternal CC; however, this finding should be considered exploratory and hypothesis‐generating. This cutoff is higher than that reported by Zhan et al.^19^ (50 mm Hg), which was derived from a population including only patients with CHD. Given the exploratory nature of this analysis and the relatively small sample size, further prospective studies are needed to determine whether PASP values in this range are consistently associated with increased maternal risk and to evaluate whether patients with mild PAH and no additional risk factors included in the CARPREG II score might have a lower risk of CC during pregnancy. Importantly, the sensitivity analysis restricted to CHD‐associated pulmonary hypertension showed similar discriminatory performance of PASP for maternal CCs, supporting the internal consistency of the main findings. Further, PASP and CARPREG II showed similar discriminatory performance for maternal CCs in this cohort. However, given the limited sample size and number of events, both the subgroup and comparative analyses should be interpreted cautiously and considered exploratory. In particular, the DeLong comparison between PASP and CARPREG II had limited statistical power due to the small number of events and should not be taken as evidence of equivalent predictive performance. In summary, the observed association between higher estimated PASP and maternal CCs might reflect overall disease severity rather than an independent predictive effect; therefore, PASP should be interpreted cautiously as a potential risk marker in this cohort.

### 4.2 Management of pulmonary arterial hypertension in pregnancy

Despite recommendations of pregnancy as a very high maternal and fetal risk in women with PAH, some proceed with gestation either electively or due to limited access to care.^21^ Our findings, and those of McNeil and Low,^20^, ^22^ highlight the importance of individualized care and timely interventions. The use of PAH‐specific therapies was limited in our cohort during pregnancy, likely reflecting resource constraints, restricted treatment availability, treatment intolerance in some patients, and the challenges of managing these medications in pregnant patients in a real‐world tertiary care setting. This underuse might have influenced maternal outcomes, as reduced access to PAH‐targeted therapy could have contributed to clinical deterioration in some cases. Although prior studies have suggested that PAH‐specific therapies might improve hemodynamic parameters and potentially influence maternal and fetal outcomes, the retrospective design and relatively small sample size of our study do not allow a definitive assessment of the effect of treatment underutilization on outcomes. Moreover, therapies used during pregnancy and the puerperium should be interpreted as markers of disease severity and escalation of care rather than independent predictors of adverse outcomes, as they were generally initiated after clinical deterioration.

The choice of anesthesia also affects outcomes. Both our study and those by McNeil^22^ and Low^20^ found that general anesthesia was more frequently used in women with complications, underscoring the need for tailored anesthetic plans to avoid hemodynamic instability.^20^, ^22^ The relatively low complication rate in our study might reflect the benefits of a proactive, multidisciplinary approach integrating cardiology, obstetrics, anesthesiology, and critical care.

### 4.3 Perinatal outcomes

Perinatal outcomes in our cohort were generally consistent with those reported in previous studies. Rates of FGR and cesarean section were comparable to those described by Ladouceur and Low.^18^, ^20^ Low et al. also noted that 76% of pregnancies resulted in cesarean section, similar to our findings. Preterm birth and NICU admissions remain significant concerns in PAH pregnancies, particularly in women with severe disease. A perinatal mortality rate of 5.5% suggests that, despite coordinated care, neonatal complications remain significant.^20^

### 4.4 Strengths and limitations

This study adds to the growing body of evidence supporting structured multidisciplinary care in PAH pregnancies. Strengths include expert echocardiographic evaluation, consistent data collection, and care in a high‐complexity referral setting. Limitations include reliance on echocardiography for PAH diagnosis and limited access to PAH‐specific therapies, which constrained direct assessment of their effects on outcomes. A further limitation is that no invasive hemodynamic assessment was performed during pregnancy; therefore, diagnosis and severity assessment in this cohort were based on echocardiographic findings and should be interpreted in that context. This is particularly relevant in patients with structural heart disease, in whom echocardiographic estimation might be less precise than invasive assessment, and because outcomes might vary according to the underlying etiology, including among different forms of CHD‐associated pulmonary hypertension. As noted by Low et al.,^20^ many studies face similar challenges, including retrospective design and variability in management approaches. Notably, part of the study period overlapped with the COVID‐19 pandemic (2020–2022), which might have influenced referral patterns, follow‐up, access to specialized care, and treatment continuity in some patients. However, no changes to clinical management were implemented for patients who received care at our center during that period. Because this study was not designed to evaluate the specific impact of the pandemic, and no period‐based comparative analysis was performed, the magnitude of any indirect effect cannot be determined; nevertheless, this should be considered a potential contextual factor when interpreting our findings.

## 5 CONCLUSION

Pulmonary arterial hypertension in pregnancy continues to pose serious health risks. However, our findings, consistent with recent evidence, suggest that maternal and perinatal outcomes might be improved through early risk assessment and coordinated multidisciplinary care. The PASP value identified in this study should be interpreted as exploratory and hypothesis‐generating, and further research is required before any specific threshold can be considered clinically actionable. Given the known risks, preconception assessment is essential for women with PAH, and individualized counseling should include a careful discussion of these risks along with all available options, including the possibility of early pregnancy termination. For women who decide to continue with pregnancy, comprehensive care in specialized centers with expertise in managing complex cardiovascular conditions is essential. Ongoing research is needed to refine risk prediction (including echocardiographic PAPS and other clinical metrics) to explore therapeutic strategies that can further support maternal and fetal health in this high‐risk population.

## AUTHOR CONTRIBUTIONS

Laura Belmont‐Rojo and Rosa Hernandez‐Cruz conceived and designed the study, supervised the clinical management of the patients, collected the data, contributed to data interpretation, and participated in drafting and critically revising the manuscript. Diana Yazmin Copado‐Mendoza contributed to the conception of the study, participated in patient recruitment and maternal–fetal clinical care, contributed to data acquisition and interpretation, and critically revised the manuscript. Ana Jimena Lopez‐Diaz participated in patient recruitment, contributed to data acquisition, assisted in drafting the manuscript, and critically revised it for important intellectual content. Tirso Rubalcava‐Rubalcava contributed to the conception and design of the study, provided specialized cardiology expertise, contributed to the interpretation of the findings, supervised the clinical aspects of the project, and critically revised the manuscript. Mario Isaac Lumbreras‐Marquez contributed to study design, performed the statistical analyses, contributed to data interpretation, drafted portions of the manuscript, and critically revised it for important intellectual content. Maria Antonia Pijuan‐Domenech contributed to study design, provided expertise in adult congenital heart disease and cardiovascular outcomes, contributed to interpretation of the findings, supervised the overall development of the project, and critically revised the manuscript. Laura Belmont‐Rojo, Rosa Hernandez‐Cruz, and Mario Isaac Lumbreras‐Marquez prepared the initial draft of the manuscript. All authors approved the final version for publication and agree to be accountable for all aspects of the work.

## FUNDING INFORMATION

This research did not receive any specific grant from funding agencies in the public, commercial, or not‐for‐profit sectors.

## CONFLICT OF INTEREST STATEMENT

None declared.

## Supporting information


**Table S1:** Medication used before and during pregnancy, and in the puerperium.
**Table S2:** Maternal cardiac complications.

## Data Availability

The data underlying this article cannot be shared publicly due to the privacy of individuals who participated in the study. The data will be shared on reasonable request to the corresponding author.
