# Supplementary material for: Maternal and perinatal outcomes of pregnant women with echocardiographically high probability of pulmonary arterial hypertension
Source: Int J Gynaecol Obstet. 2026 Jun 18;175(3):1296–304. doi: 10.1002/ijgo.71119 (PMC13769352; doi:10.1002/ijgo.71119)
Supplement: Supplementary file 1 [file IJGO-175-1296-s001.docx]

**Table S1. Medication used before and during pregnancy, and in the puerperium.**

| **Parameter** | **Full cohort** | **Without cardiac complications** | **With cardiac complications** | ***P*-value** |
| --- | --- | --- | --- | --- |
|  | **N = 62** | **N = 51** | **N = 11** |  |
| Medications used **before pregnancy**, n (%) |  |  |  |  |
| Anticoagulants | 1 (1.6) | 1 (2.0) | 0 (0.0) | 0.999 |
| Antihypertensive drugs | 2 (3.2) | 1 (2.0) | 1 (9.1) | 0.326 |
| Antiarrhythmic drugs | -- | -- | -- | -- |
| Pulmonary vasodilators | -- | -- | 1 | -- |
| Diuretics | -- | -- | -- | -- |
| Beta blockers | 1 (1.6) | 0 (0.0) | 1 (9.1) | 0.177 |
| Inotropic drugs | -- | -- | -- | -- |
| Medications used **during pregnancy**, n (%) |  |  |  |  |
| Anticoagulants | 8 (12.9) | 4 (7.8) | 4 (36.4) | 0.027 |
| Antihypertensive drugs | 1 (1.6) | 0 (0.0) | 1 (9.1) | 0.177 |
| Antiarrhythmic drugs | -- | -- | -- | -- |
| Pulmonary vasodilators | 4(6.5) | 3 (5.9) | 1 (9.1) | 0.552 |
| Diuretics | 5 (8.1) | 1 (2.0) | 4 (36.4) | 0.003 |
| Beta blockers | -- | -- | -- | -- |
| Inotropic drugs | -- | -- | -- | -- |
| Medications used in the **puerperium**, n (%) |  |  |  |  |
| Anticoagulants | 8 (12.9) | 4 (7.8) | 4 (36.4) | 0.027 |
| Antihypertensive drugs | -- | -- | -- | -- |
| Antiarrhythmic drugs | 3 (4.8) | 3 (5.9) | 0 (0.0) | 0.999 |
| Pulmonary vasodilators | 25 (40.3) | 16 (31.4) | 9 (81.8) | 0.005 |
| Diuretics | 14 (22.6) | 3 (5.9) | 11 (100.0) | <0.001 |
| Beta blockers | 10 (16.1) | 3 (5.9) | 7 (63.6) | <0.001 |
| Inotropic drugs | 4 (6.5) | 0 (0.0) | 4 (36.4) | 0.001 |

**Table S2. Maternal cardiac complications.**

| **Case** | **Age (years)** | **Etiology** | **CARPREG II** | **SPAP (mmHg)** | **LVEF (%)** | **Mode of delivery** | **FGR** | **GA at delivery (weeks)** | **Complication** | **Maternal death** | **Time of death or complication** |
| --- | --- | --- | --- | --- | --- | --- | --- | --- | --- | --- | --- |
| 1 | 19 | PAH | 5 | 102 | 30 | Instrumental vaginal delivery (forceps) | Yes | 38 | Heart failure, arrythmia and pulmonary embolism | Yes | 7 days postpartum |
| 2 | 31 | AVSD, severe insufficiency left AV valve | 5 | 96 | 55 | Perimortem  C-Section | Yes | 30 | Heart failure and pulmonary embolism | Yes | Pregnancy 30 weeks |
| 3 | 40 | ASD and VSD | 5 | 98 | 56 | C- section | Yes | 30 | Heart failure | Yes | 1 day postpartum |
| 4 | 17 | DORV | 5 | 46 | 58 | C- section | Not | 33 | Heart failure | Yes | 6 days postpartum |
| 5 | 18 | VSD and Einsenmenger Syndrom | 5 | -- | 65 | Dilatation and curettage | -- | 18 | Hear failure | No | 2 days postpartum |
| 6 | 20 | AVSD, single AV valve with severe insuficency | 3 | >65 | 50 | Vaginal delivery | Yes | 27 | Heart faillure | No | 1 day postpartum |
| 7 | 28 | Truncus arteriosus I | 3 | 100 | 58 | C- section | Yes | 36 | Heart faillure | No | 5 days postpartum |
| 8 | 25 | VSD | 2 | 66 | 50 | C- section | Yes | 35 | Heart faillure | No | 1 day postpartum |
| 9 | 26 | ASD | 3 | 70 | 47 | C- section | No | 38 | Heart faillure | No | 4 days postpartum |
| 10 | 20 | ASD | 3 | 77 | 50 | C- section | No | 37 | Heart faillure | No | Pregnancy 34 weeks |
| 11 | 28 | TGA with atrial switch | 3 | 100 | 43 | C- section | No | 39 | Heart faillure | No | Pregnancy 25 weeks |

SPAP = systolic pulmonary artery pressure; LVEF = left ventricular eyection fraction; FGR = fetal growth restriction; GA = gestational age; PAH = pulmonary arterial hypertension; AVSD = atrioventricular septal defect; AV = atrioventricular; LV = left ventricular; ASD = atrial septal defect; VSD = ventricular septal defect; DORV = Double outlet right ventricle; TGA = transposition of the great arteries
